# My Vision of Electric-Field-Aided Chemistry in 2050

**DOI:** 10.1021/acsphyschemau.3c00064

**Published:** 2024-02-01

**Authors:** Sason Shaik

**Affiliations:** Institute of Chemistry, The Hebrew University of Jerusalem, Jerusalem 9190401, Israel

**Keywords:** Diels−Alder Reactions, Electric Tweezers, Electrostatic Effects, Oriented-External Electric Fields (OEEFs), pH Switches, Reaction Axis, Water Droplets

## Abstract

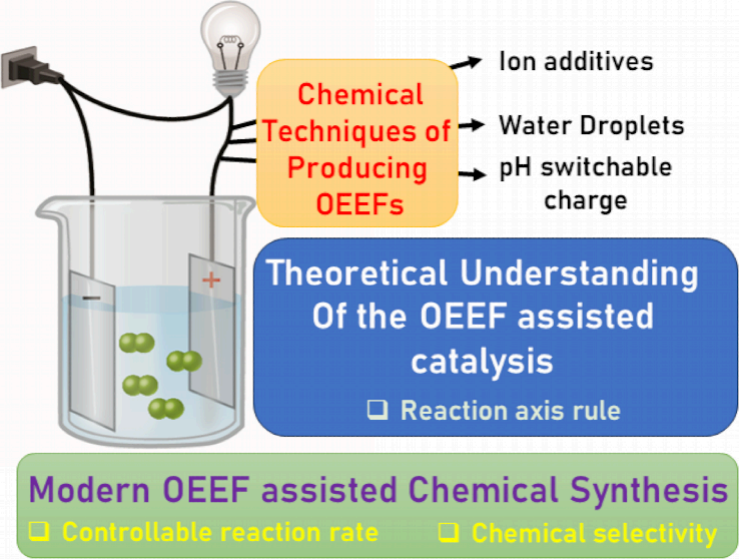

This manuscript outlines my outlook on the development of electric-field (EF)-mediated-chemistry and the vision of its state by 2050. I discuss applications of oriented-external electric-fields (OEEFs) on chemical reactions and proceed with relevant experimental verifications. Subsequently, the Perspective outlines other ways of generating EFs, e.g., by use of pH-switchable charges, ionic additives, water droplets, and so on. A special section summarizes conceptual principles for understanding and predicting OEEF effects, e.g., the “reaction-axis rule”, the capability of OEEFs to act as tweezers that orient reactants and accelerate their reaction, etc. Finally, I discuss applications of OEEFs in continuous-flow setups, which may, in principle, scale-up to molar concentrations. The Perspective ends with the vision that by 2050, OEEF usage will change chemical education, if not also the art of making new molecules.

## Introduction

Attempting to predict the future of chemistry in about 26 years is probably presumptuous. Partly, this is because chemistry has generally developed from occasional discoveries of ingenious experimental results (e.g., the Diels–Alder reaction, the discovery of isomerism, of chirality, etc.), which have determined the future course of our science. Chemistry will continue to develop in this manner.

Nevertheless, I wish to try and paint here a future for a particular topic^1−5^ which I helped shape and which has largely emerged from the interplay of theory and experiment. The theoretical prediction of using oriented external electric fields (OEEFs)^1−4^ to control chemical reaction rate and selectivity has already been followed by experimental results and techniques, which have *the potential of setting a new future of chemical synthesis*(5−14) and, hence, of chemistry as a whole. I can already see this future glimpsing from the corner...

This Perspective paints a future vision of chemistry. Section 7 outlines principles for understanding and predicting OEEF effects on chemical reactivity. A key principle is the “reaction-axis rule”, which defines the electric field direction that will most effectively enhance the reaction rate. Since reactions involve transformations of the electron pairing modes of the reactants to those of the products, the use of curly arrows will define the reaction axis (Scheme 4). Another key point in Section 7 is the interaction energy (eq 1) between the OEEF (*F*) and the molecular dipole moment (μ) vectors. Using this equation demonstrates that the OEEF acts as tweezers (Scheme 3) that orient the molecules along the reaction axis and enable their reaction. Guided by these principles, this short Perspective article summarizes past achievements in the field and then outlines potentials for future developments in harnessing OEEFs in the service of advancing chemistry.

The area of electric fields in enzymes, which has been discussed elsewhere,^15−17^ will not be covered here. Most of the following text is written in personal style as a story about sparking the development of a new area in chemistry.

## 1 The OEEF Concept: Its Story of Conception

My interest in the topic dates back to the 1970s during my Ph.D. studies at the University of Washington (in Seattle). I attended a course in the first year (1974), in which our teacher, the late Yeshayau Pocker, described a million-fold catalysis of heterolytic bond rearrangement reactions using high concentrations of LiClO_4_ in diethyl ether (5–6 M).^18^ Ever since then, studying the impact of external electric fields on chemical reactions has become an obsession that still captivates me.

During my postdoctoral year (1978–9) with Roald Hoffmann at Cornell, I started thinking about the derivation of selection rules for applying OEEFs to chemical reactions and structures. I used to discuss these ideas with my peers, Eluvatingal D. Jemmis and Al Pinhas. We calculated some simple molecules (e.g., formaldehyde) in the presence of electric fields along the three space axes. But, these initial calculations did not give rise to a reactivity picture. It was clear to me then that this idea may require extensive development before it can constitute a general approach for chemical synthesis and structural manipulation by applying OEEFs.

In the end of my postdoctoral year, I joined the faculty at Ben-Gurion University in Beer-Sheva (Israel). I was intent on analyzing the OEEF effects as soon as I could. But this was delayed for a compelling reason: I first had to understand how to model chemical reactivity... Luckily, while at Cornell, I started developing a procedure that maps molecular orbital (MO) wave function (without and with configuration interaction) into valence bond (VB) wave functions. The application of this mapping procedure for simple reactions along the entire reaction coordinate resulted in the development of VB diagrams for chemical reactivity. The entire story was published in 1981^19^ and has been followed by applications and reviews, e.g., ref (20).

In mid-1992, I joined Hebrew University. During the initial years in Jerusalem, I focused on applications of VB diagrams with my first Ph.D. student, Avital Shurki,^20^ and on chemical bonding with my postdoc Johnny Galbraith.^21^ Simultaneously, I have gotten engaged in intense collaborations on chemical bonding,^22,23^ with Philippe Hiberty and the members of the “VB community”, which included among others, my research assistant David Danovich, and Wei Wu (from Xiamen University) who originated the friendly VB software (XMVB), and came for one year to Jerusalem to apply VB to chemical problems. *The VB activity made me realize the importance of ionic structures for bonding and reactivity, especially in the presence of electric fields*.

In retrospect, the acquired VB thinking turned out to be essential for the developments of the current notions of OEEF and formulating the “reaction axis rule”, which defines the best action of OEEFs on structure and reactivity.^1−4^ The “reaction axis” is the essential direction along which the OEEF should be applied in order to enhance (or inhibit, if so desired) reaction rates. This rule has served as a conceptual guide and proved to be useful and easily applicable.^3,4^ As reviewed by Siddiki et al.,^17^ by now, the task of locating the directions of OEEFs that will minimize the energy barrier for a given reaction system have also been implemented in computational procedures, for example, in refs (24−26). My primary goal in the present Perspective is to outline key principles of using OEEF and to note a few recent experimental studies, which may impact the future of mainstream chemistry and, hence, also my vision for 2050.

## 2 Theoretical Predictions of OEEF Applications

**Figure 1:**
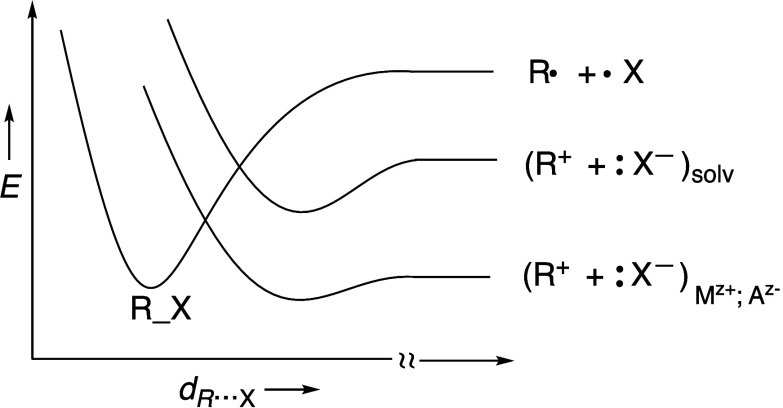
Bond heterolysis in a solvent is due to crossing of the covalent and ionic curves along the bond stretching coordinate. An ion pair, M^z+^:A^z–^, which creates an oriented electric field, leads to rate enhancement. The Figure is reproduced with permission from ref (20), Figure 9. Copyright (1999) Wiley VCH.

The OEEF research bore initial fruits in the 1990s,^20^ when Shurki and I analyzed, among other topics, the electrostatic catalysis of heterolytic bond cleavage by an OEEF of a “salt molecule” made of a pair of oppositely charged ions. This electrostatic rate enhancement is qualitatively illustrated in Figure 1 as the lowering of the crossing point between the covalent and ionic curves along the bond stretching reaction coordinate.

**Figure 2:**
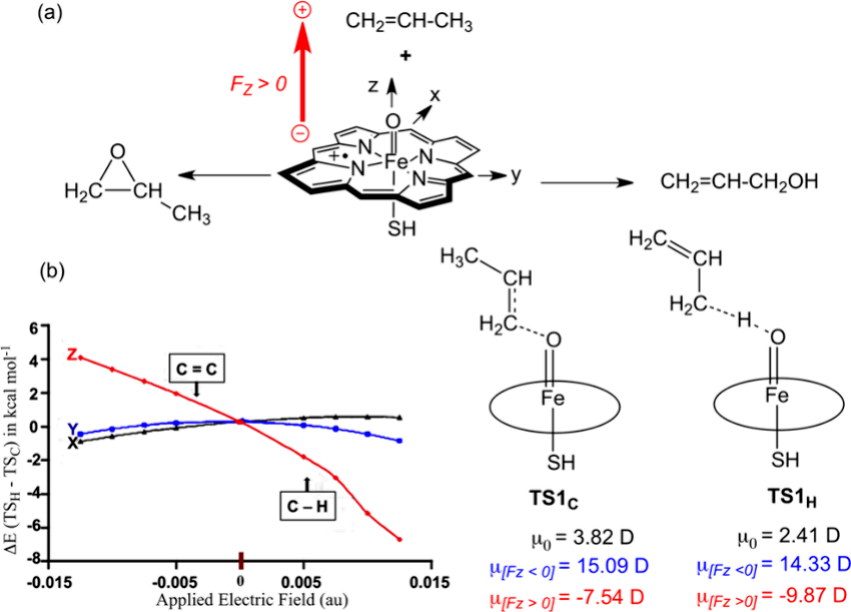
(a) C–H hydroxylation vs C=C epoxidation of propene in the presence of OEEFs (in au; 1 au = 51.4 V/Å) along the three Cartesian axes. F_Z_ points along the Fe=O bond of Compound I. The conventions for positive and negative F_Z_ vectors follow, here and elsewhere in the text, the Gaussian software. (b) The direction of F_Z_ determines the regiochemical preference: **F**_**Z**_**< 0 prefers C=C epoxidation, while F**_**Z**_**> 0 prefers C–H hydroxylation**. Shown on the right-hand side are the dipole moments (in Debye units, D) for the initial states (μ_0_) and in the respective TSs, for the two directions of F_Z_. Note that F_X_ and F_Y_ have hardly any effect on either reactivity or product selectivity. The Figure is adapted with permission from ref (4), Figure 3. Copyright (2016) Nature Publishing Group.

This analysis prompted me eventually in 2004 to compute OEEF effects on the selectivity of C–H/C=C oxidation of propene by a model of the active species of Cytochrome P450, shown in Figure 2a and called “Compound I”.^3,4,27^ Together with my postdoctoral fellows, de Visser and Kumar, we found^27^ that the oxidation process responds to the OEEF in a directionally selective manner. It occurs preferentially when the OEEF is oriented along the *Z*-axis (Figure 2b) in the directions of the Fe–O···H-C or Fe–O···C=C moieties. Thus, the application of the OEEF along F_Z_ lowered the reaction barriers, in a manner that depends on the F_Z_ orientation. A negatively oriented F_Z_ led to preferential C=C epoxidation, while simply flipping the OEEF to a positively oriented direction led to preferential C–H bond hydroxylation. The corresponding transition state (TS) dipole moments (μ) in Figure 2b show that in both F_Z_ directions, the dipole moments of the respective TSs are increased significantly by the OEEF. Note that the F_X_ and F_Y_ OEEFs have little effect on the product selectivity. Thus, the OEEF is strictly selective! **In the absence of the OEEF, there is no product selectivity**.

This reactivity pattern of Compound I revealed that Nature must be an avid employer of OEEFs, as is indeed apparent in a variety of natural enzymes.^15−17^ In subsequent theoretical studies of other chemical reactions (e.g., Diels–Alder^28^ and S_N_2^29^ reactions), *OEEFs emerged as efficient effectors of chemical change and the future smart reagents of chemistry*.^3,4^ I was convinced that OEEFs usage must be exported to the chemists’ bench!

## 3 Experimental Tests of Theoretical Predictions

**Figure 3:**
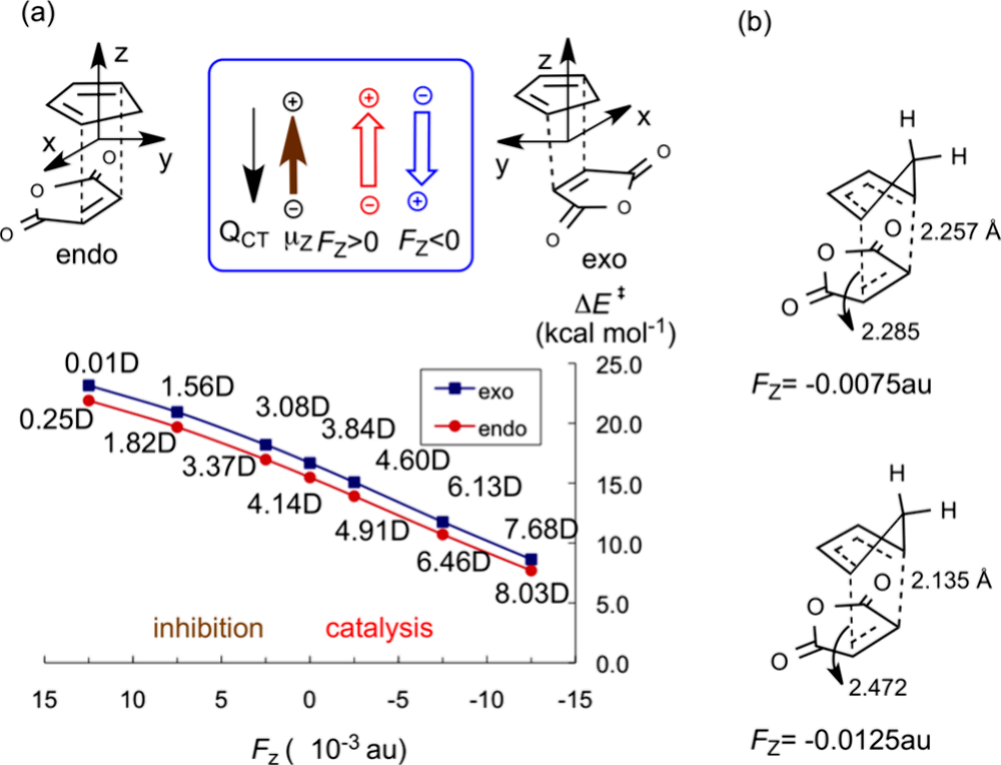
(a) The *endo*- and *exo*-TS structures for the Diels–Alder reaction of cyclopentadiene and maleic anhydride. Underneath the structures are plots of barrier heights as a function of F_Z_ (F_X_, F_Y_ have virtually no impact on the barrier height). (b) The TS structures on the right-hand side show that as F_Z_ becomes more negative, the asynchronicity of the two forming C–C bond lengths in the TS increases and leads eventually to zwitterionic intermediates. The Figure is adapted with permission from Figure 6 in ref (4). Copyright (2016) Nature Publishing Group.

In those days, theoretical predictions in chemistry were still taken with a grain of salt, especially if the prediction seemed to require unusual or not easily available tools to be experimentally tested. A turning point was the 2010 paper,^28^ in which we showed that the rate enhancement of the Diels–Alder (DA) reaction responds to the OEEF vector in a unidirectional manner, along the Z direction. By contrast, the fields along X and Y do not affect the barrier in any significant manner. The results are summarized in Figure 3 for cyclopentadiene reacting with maleic anhydride, under an F_Z_ OEEF, which varies from negative to positive. Thus, Figure 3a shows that a negatively oriented F_Z_ < 0 lowers the energy barrier by ∼7 kcal/mol and increases the dipole moment of the TS by about 4 D (indicating increased mixing of ionic structures). By contrast, flipping the field’s direction to F_Z_ > 0 raises the barrier and lowers the TS-dipole moment by similar amounts. Furthermore, as the negatively oriented OEEF (F_Z_ < 0) increases in magnitude, the respective TS structures (Figure 3b) become bond-asymmetric, and eventually the reaction generates a zwitterionic intermediate.^28^ Note that rate acceleration and inhibition occur even at very small F_Z_ values.

**Figure 4:**
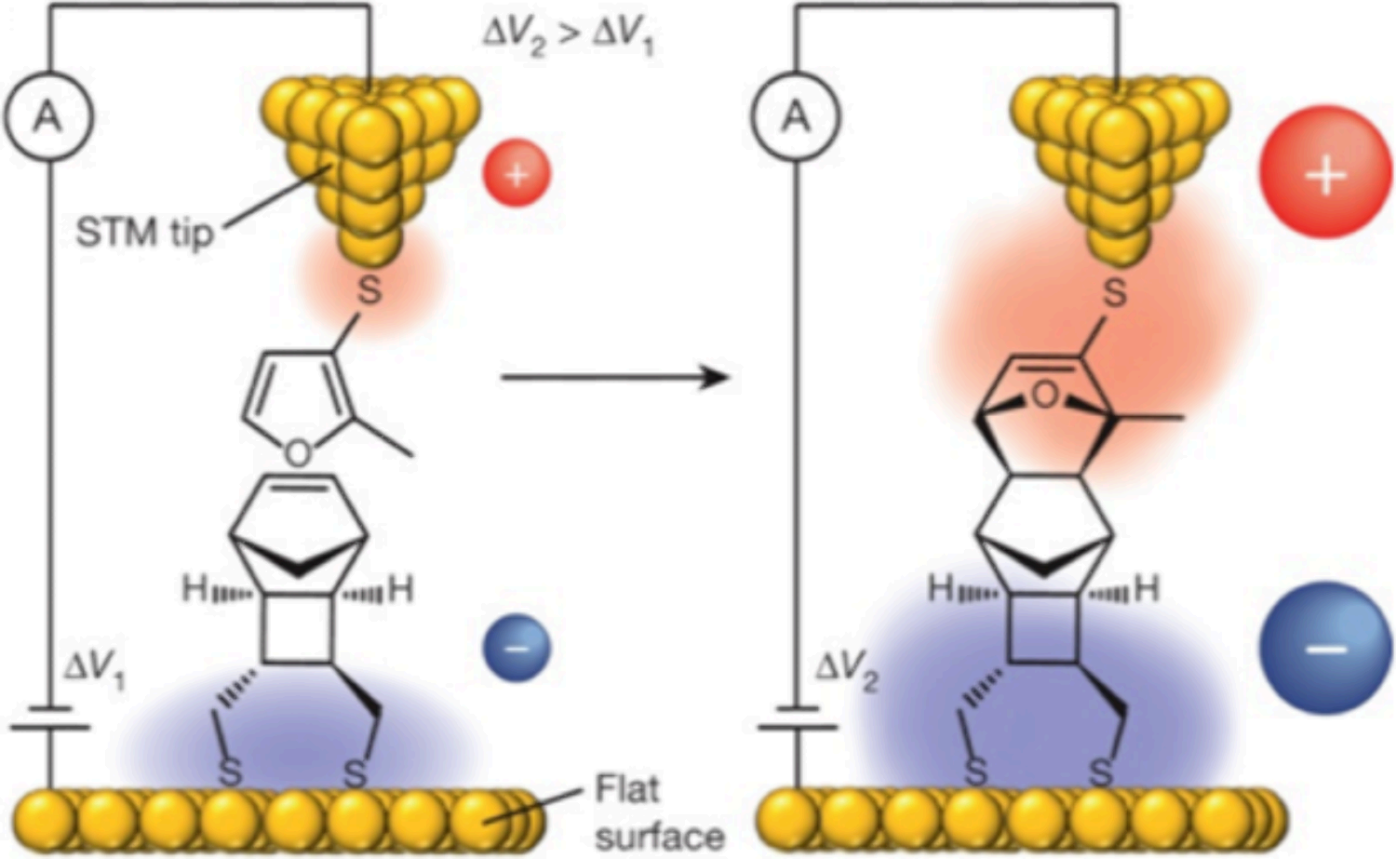
An experimental setup for testing the prediction of the OEEF effect on a Diels–Alder reaction. The setup employs an STM tip and a gold surface that orient the reactants along the OEEF vector created by the voltage gauge. The product formation event was verified by monitoring the current flow as the adduct was formed and by breaking the junction between the STM tip and the adduct, that stop the current. The Figure is adapted with permission from ref (3). Copyright (2018) of Royal Society of Chemistry.

Before its acceptance for publication, the DA manuscript^28^ had a hard time with leading journals. Nevertheless, in 2016, six years after its publication, the manuscript attracted the interest of a group of experimentalists from Spain, England, and Australia,^6^ who joined forces and created a beautiful design for testing the predictions on the DA reaction. As shown in Figure 4, the group utilized a diene molecule linked to an STM tip and a dienophile, linked to a gold electrode. Using this setup, the group solved elegantly the issues of orienting the reactants, while simultaneously delivering a unidirectional OEEF pulse that brought about an acceleration of the reaction rate only in a single direction (further technical details are available from the authors of ref (6)).

The above verification of the selective directionality of the rate enhancement of the DA reaction was further supported in similar experiments by Huang et al.^12^ and Yang et al.^13^ Thus, Huang et al.^12^ used STM-tip/Au-electrode to probe a two-step reaction mechanism, in which the reaction axes of the steps were mutually orthogonal. They demonstrated that the only step that was affected by the OEEF is the one in which the reaction axis had a component along the direction of the OEEF. In the experiment of Yang et al.^13^ a maleimide dienophile was linked to fluorescein (serving as sensor), which in turn was connected to graphite electrodes. At the same time, the furan dienes were allowed to find their ways to the dienophile and react with it. This setup shows that **fixing the reactants in a reacting-pose is not absolutely necessary** for reactions under OEEF (more on this issue later). Furthermore, Yang et al.^13^ confirmed also the formation of a zwitterionic intermediate at the extreme voltage range used in their experiment.

Various other techniques for creating OEEFs and changing their directions were discussed by Ciampi et al.^5^ Let me illustrate two additional techniques that exploit the power of OEEF to accelerate/inhibit reactions at will.

**Figure 5:**
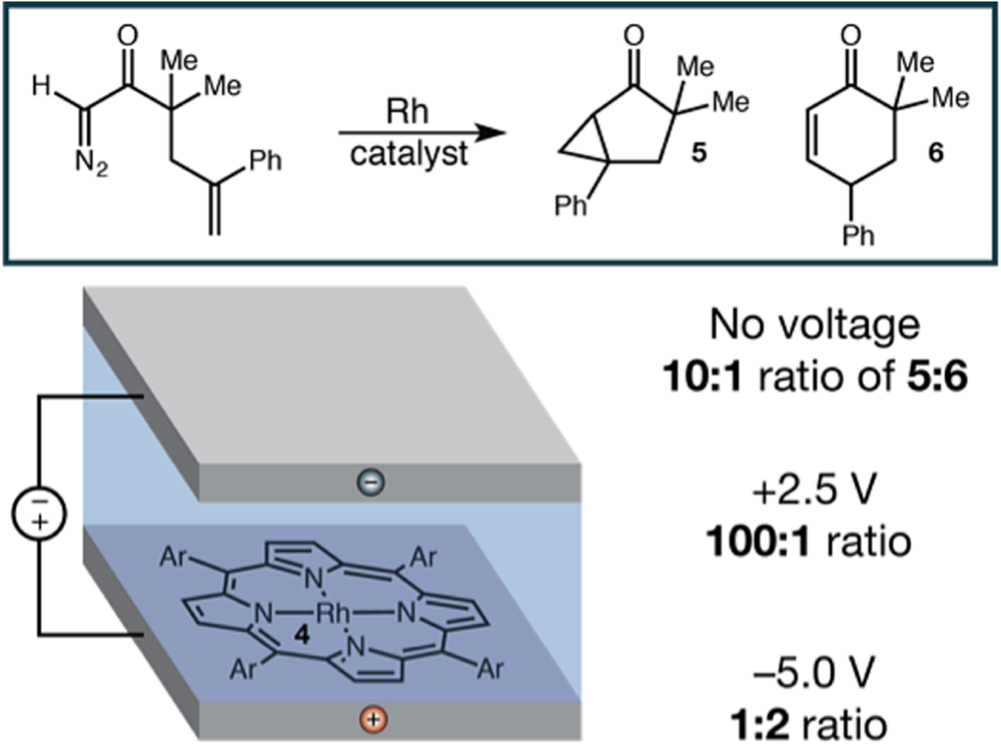
OEEF, due to the ion separation in the double-layer of an electrochemical cell (without a Faradaic current), controls the product-selectivity of the rhodium-porphyrin-induced rearrangement of the azo compound. The Rh complex is linked to an electrode coated by an insulator that prevents a Faradaic current flow. The voltage and its sign are seen to affect the product-selectivity ratio **5**/**6**. The Figure is adapted with permission from ref (4), Figure 19. Copyright (2016) Nature Publishing Group.

Figure 5 shows the work of Kanan et al.,^11^ on harnessing the double-layer of an electrolyte in an electrochemical cell. The Rh complex in the cell is linked to the electrode, which is coated by an insulator that blocks any Faradaic current flow. As such, the separation of the oppositely charged ions of the electrolyte creates an OEEF in the cell. In turn, the OEEF affects the product selectivity of the carbene rearrangement induced by the rhodium-porphyrin complex. It is seen that the ratio of products **5**/**6** is reversed, from >100:1 all the way to 1:2, depending on the magnitude and direction of the applied voltage bias. Changing the voltage sign from positive to negative inverted the product-selectivity ratio. According to Kanan, in his subsequent experiments, upscaling the method to generate larger product quantities encountered technical difficulties. Further discussion of ion distributions in the double-layer can be found in the chapter by Ciampi et al.^5^

The above techniques use OEEFs that are external to the reacting systems. Another known technique generates electric fields by employing molecular species, which can create an OEEF upon polarization or charging. Let us discuss some of these methods.

**Figure 6:**
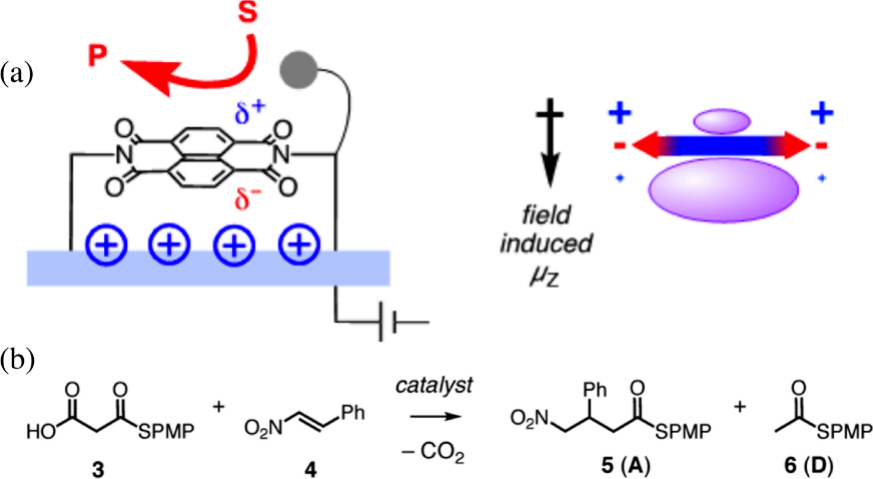
(a) OEEF generation due to surface charging by an applied voltage. The surface charging polarizes the π-anion catalyst, which develops a dipole (μ_Z_) and, hence, a corresponding OEEF. Note that here, the direction of the dipole moment follows chemical convention wherein the arrowhead is the negative pole. (b) The OEEF prefers the production of **6(D)**, while in the absence of OEEF, the major product is **5(A)**. The Figure is adapted from ref (7). Copyright (2017) American Chemical Society.

Figure 6 shows one such technique, which is used by Matile et al.^7^ This group employs surface charging, which induces charge polarization of a π-anion catalyst and hence an OEEF (Figure 6a). The Figure illustrates the polarization of the π-anion catalyst and a nascent dipole moment. In turn, the dipole moment is attended by an OEEF, which affects the product selectivity of the complex reaction in Figure 6b. Thus, in the absence of surface charging, the major product is **6(D)**. However, application of the surface charging changed the major product to **5(A)**, which possesses the larger dipole moment in its transition state^7^ and, hence, enjoys greater stabilization by the induced OEEF of the π-anion catalyst. In principle, changing the direction of the voltage would have flipped the OEEF direction and might have affected the product selectivity in a different manner. This however was not yet tried by the Matile group.

## 4 Other Tests Using Oriented Internal Electric Fields (OIEFs)

An ingenious approach to electrostatic catalysis was outlined by Coote and her co-workers using molecular species which undergo charging by a change of the pH and, hence, create oriented internal electric fields (OIEFs).^5,14^ The method is termed **a pH switch**.

**Figure 7:**
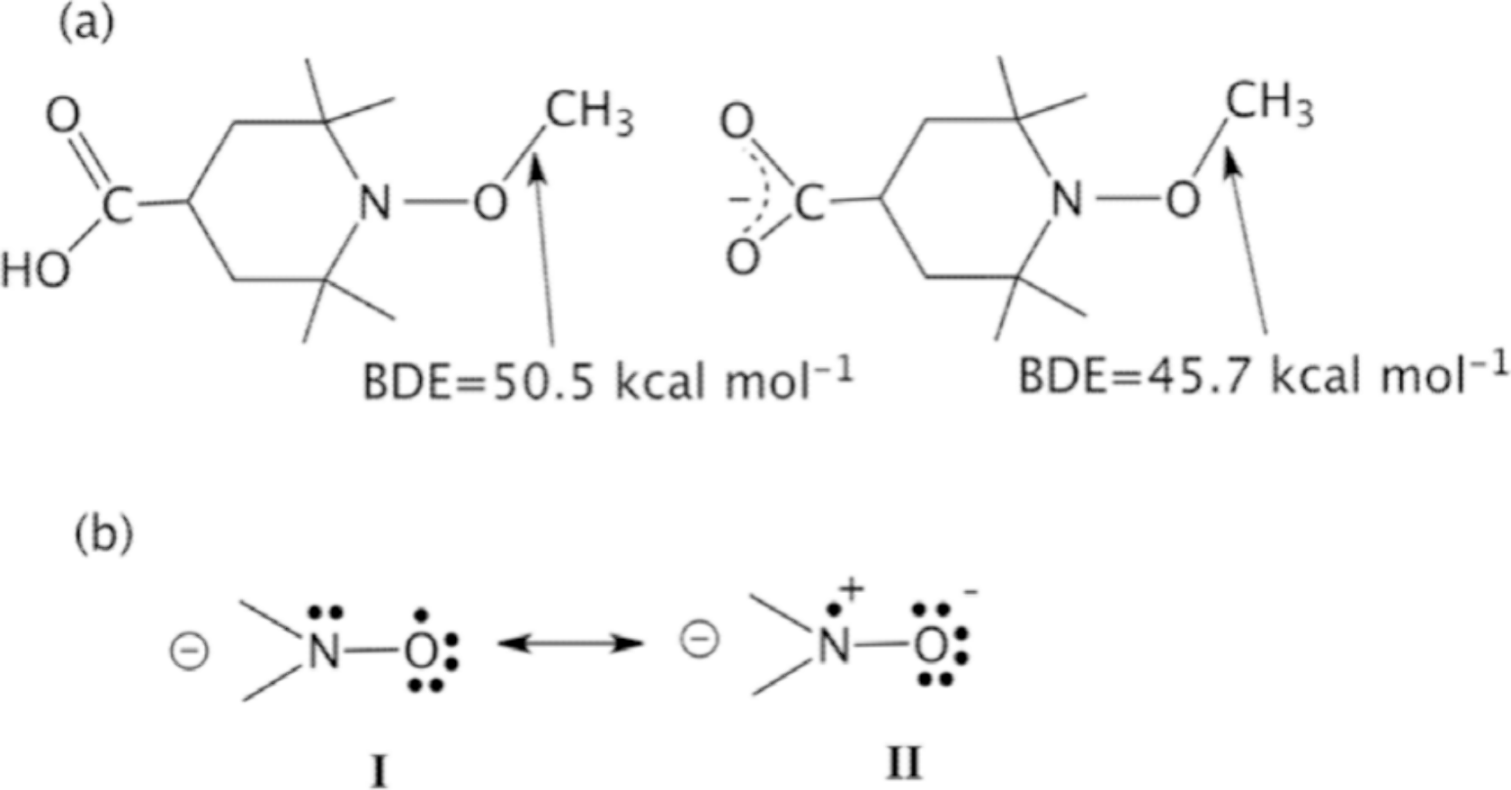
(a) TEMPO derivatives and corresponding changes in the BDE values (kcal/mol) of O–CH_3_ due to the pH switch that deprotonates the carboxylic acid substituent (−CO_2_H). (b) Stabilization of the nitroxyl radical by the enhancement of resonance structure **II**, due to the OIEF effect of the negative charge. The Figure is reproduced with permission from ref (3), Scheme 6. Copyright (2018) Royal Society of Chemistry.

The impact of pH switching is illustrated in Figure 7, for the TEMPO (tetramethylpiperidine-1-oxyl) derivative. Figure 7a shows the effect of switching a negative charge on the remote O–C bond energy. Thus, compared to the neutral molecule, the O–C bond dissociation energy of the negatively charged TEMPO derivative is reduced by approximately 5 kcal/mol.^30^

Figure 7b shows that the negative charge near the nitroxyl radical moiety (that is generated by O–CH_3_ cleavage in Figure 7a) enhances the contribution of resonance structure **II** and affects the nitroxyl properties. For example, the OIEF reverses the relative energy levels of the highest doubly occupied and singly occupied molecular orbitals of the nitroxyl radical^30,31^ and stabilizes the radical by increasing its delocalization.

The OIEF due to pH switching also affects the kinetics and thermodynamics of H atom transfer reactions,^32^ radical-mediated polymerization reactions,^33^ and CO_2_ storage by the carboxylation of *o*-alkylphenyl ketones.^34^ This latter study by Blyth and Coote^34^ was conducted also by experimental means.

**Scheme 1:**
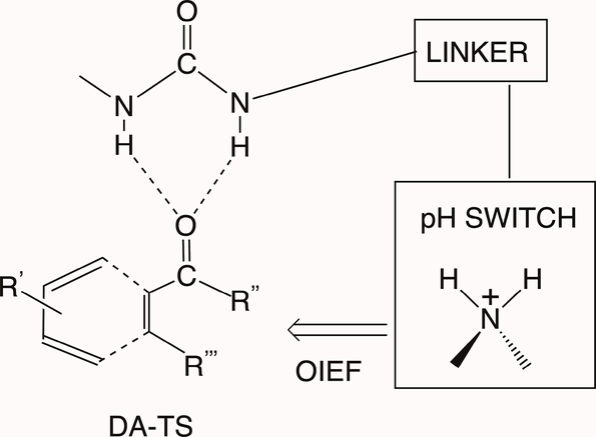
A Schematic Sketch of a Molecular Setup, Which Is Used in Ref (14), to Enhance the Rate of the Diels-Alder (DA) Reaction The setup includes a built-in ammonium group, which is generated by a pH switch, and which thereby exerts an OIEF that lowers the energy of the DA-TS. The scheme was designed and drawn by the author of this Perspective.

Blyth and Coote^14,35^ designed also a pH-switch that catalyzes Diels–Alder reactions. As sketched in Scheme 1, the setup involves a short hydrogen-bond linker, which “holds” the Diels–Alder transition state (DA-TS), and a charged ammonium group (>H_2_N^+^) which is created by pH switching. In turn, this charged group generates an OIEF that stabilizes the DA-TS and accelerates thereby the reaction between an enone and a diene. Blyth and Coote^14^ found by computational means that the switching of the OIEF lowers the reaction barriers for various reactions by 2.4–7.6 kcal/mol. These barrier lowering effects were reduced in solvents but did not vanish, even in acetonitrile.

## 5 Use of Ionic Additives to Accelerate Reactions and Affect Mechanisms

Ionic additives have significant electric fields, which lower energy barriers and affect reaction mechanisms.^36,37^ For example, Clark^36^ showed that a Li^+^ cation favors the addition of, e.g., methyl radical to propene double bond over the allylic H-abstraction. Clark concluded that the electrostatic catalysis elicited by Li^+^ is responsible for the remarkable finding that Li^+^ salts induce polymerization of simple terminal olefins.^38^

Another case of electrostatic catalysis was discovered by Li, Schwarz et al.,^37^ using a combination of gas-phase experiments followed by computations. Thus, Cu^+^*promotes a concerted and barrier-free insertion of a C atom into two C–H bonds of CH*_*4*_ to yield a Cu^+^ coordinated ethylene, Cu^+^(C_2_H_4_). The reaction is mediated by the singlet state of CuC^+^, which crosses below the corresponding triplet state at a long Cu–C distance. Modeling of the reaction^37^*showed that the effect of Cu*^*+*^*is reproduced by using an electric field of a point-charge model*. By contrast, the mechanism of this reaction changes in the presence of Au^+^. The Au^+^ cation forms covalent bonding with C and, thereby, induces a stepwise mechanism during the formation of Au^+^(C_2_H_4_).^37^

Similarly, Groves et al.^39,40^ showed that substituting the outer circumferences of the porphyrin rings of a model of Compound I and its Fe^III^–OH derivative, with positively charged rings (e.g., *para*-methylated pyridines), accelerates the rates of their H-abstraction reactions by several orders of magnitude, reaching million-fold. Our computational analysis^41^ showed that the charged porphyrin rims create electric fields (on the oxo/hydroxo moieties of Fe=O/Fe^III^–OH), which elicit electrostatic acceleration of the H-abstraction reaction.

## 6 Use of Water Microdroplets to Accelerate Reactions

**Scheme 2:**
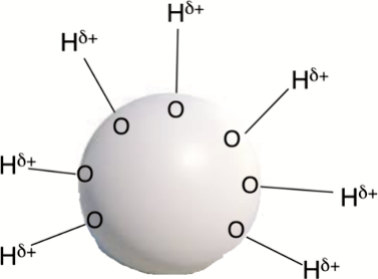
A Cartoon Representation of a Water Droplet and Some of Its On-Surface External O–H Bonds These bonds exert electric fields which affect chemical reactivity. The scheme was designed by the author of the Perspective.

Zare et al.^42,43^ showed that interfaces of water–air, water–solid, and water–oil generate water droplets which give rise to H_2_O_2_ and other unusual products. Recently, Laage et al.^44^ used molecular dynamic simulations and demonstrated that the droplet surface is acidic and contains excess H_3_O^+^ ions. As such, the droplet surface generates a unique source of molecular electric field,^45^ which accelerates nucleophilic-cleavage reactions.^10^ As such, this accelerated nucleophilic cleavage reactivity originates in an oriented electric field, which can be conveniently represented by the cartoon in Scheme 2. The cartoon shows external O–H bonds that are perpendicular to the droplet surface and which carry significant positive charges on the H atoms and exert thereby an oriented electric field capable of accelerating chemical reactions.

Head-Gordon et al.^46^ used computational modeling to show that the on-surface O–H bonds have significant electric fields. Furthermore, they found that the droplet surface electric fields of ∼0.16 V/Å yield enough power to lower the activation energy and accelerate the rates of chemical reactions by several orders of magnitude. Indeed, Nandi et al.^47^ found that water droplets can generate aryl carbocations from phenols by an aromatic S_N_1 reaction, which is presumably accelerated by the protonation capability of the droplet. More recently, Zhang, Xie et al.^48^ reported that water droplets accelerate, by 7 orders of magnitude, the Menshutkin S_N_2 reaction between pyridine and CH_3_I. Further impact of the O–H electric fields was discussed for the mechanism of ammonium hydroxide formation in water.^49^

## 7 Principles for Understanding/Predicting OEEF Effects

Let me proceed with a few principles that are helpful to keep in mind, during the application of OEEF to chemical reactions:

a The first one is the reaction axis rule.^2−4^ For convenience, we shall uniformly label the reaction axis as Z, and hence the OEEF will correspond to the vector F_Z_. Once we defined the reaction axis, we also specified the direction that will most effectively enhance the reaction rate. By contrast, flipping the direction of the OEEF vector along the same axis will inhibit the reaction. As we shall see next, locating the reaction axis is simple and extremely intuitive. At the same time, it is optional to use one of the computational methods, which have been developed to locate the OEEF direction which will optimize the barrier-lowering effect.^24−26^ Similarly, there are methods which calculate electric fields from charge distributions.^17,50,51^
b Application of the F_Z_ OEEF along the reaction axis of a given reaction will polarize the TS and increase its dipole moment (μ_Z_), as shown in Figure 2b and Figure 3a. Flipping the direction of the OEEF along the same axis will decrease the dipole moment of the TS (e.g., see Figure 3a).
c The interaction energy of the OEEF, with the polarized dipole moment of the TS or of any species along the reaction axis, is given by eq 1, as a direct product of the OEEF vector (F_Z_ in units of V/Å) and the corresponding molecular dipole moment vector (μ_Z_ in Debye (D) units) which in turn, involves the due polarization^17^ by the OEEF.

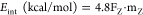
1

**Scheme 3:**
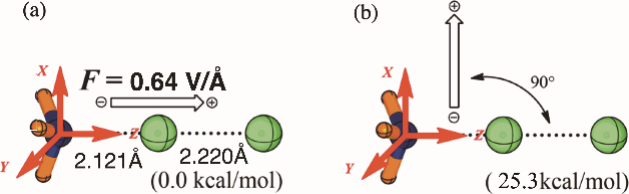
Computed Interaction Energy of the OEEF (F_Z_ = 0.64 V/Å) with the Halogen Bond H_3_N–Cl_2_ is 25.3 kcal/mol Data from the respective supporting document (Table S35) of ref (53). Adapted from ref (53). Copyright (2019) American Chemical Society.

Generally, because the TS is usually the most electronically delocalized and polarizable species along the reaction path, the maximal impact of F_Z_ is on the TS species of a chemical reaction. Using a VB language, application of the OEEF enhances the mixing of ionic structures into the wave function of the TS.^4,28^ This affects thereby the resonance energy stabilization of the TS and its dipole moment and, hence, also the corresponding interaction with the electric field (eq 1).^4,17,28^ Indeed, as we showed in Figure 3, application of the OEEF in one direction increases the dipole moment of the respective TSs, and its corresponding interaction energy is shown in eq 1. Therefore, the OEEF lowers energy barriers significantly when F_Z_ is aligned along the reaction axis. Flipping the direction of F_Z_ along the reaction axis will decrease the TS’s dipole moment and raise the barrier (see Figure 3). Since eq 1 involves a direct product of the electric field and the molecular dipole moment, the largest impact will be when the two vector quantities are colinear, i.e., along the reaction axis (Z).

d It is often said that a major problem for implementing the usage of OEEFs is the requirement to orient the reactants along the OEEF. However, this problem does not really exist **since the OEEF itself will orient the molecular complex in space**, by increasing its dipole moment and optimizing the interaction energy with the OEEF.^52−54^ Thus, for example, the interaction energy of the OEEF and the halogen bond between NH_3_ and Cl_2_, in Scheme 3, is 25.3 kcal/mol.^53^ The formation of the halogen bond complex H_3_N–Cl–Cl is followed by a barrier of 21.8 kcal/mol for the Cl–Cl bond cleavage by the base. As such, the OEEF acts as tweezers^52−54^ that capture the molecular complex and prepare it for the respective reaction.^53^

e The interaction energy of F_Z_ with the TS dipole-moment is affected by the presence of solvents.^55^ Polar solvent molecules undergo arrangement in the presence of the OEEF and screen thereby its effect on the reacting system. Nevertheless, in reactions which are typified by large charge-shift (and hence, also a large TS dipole), e.g., as in the Menshutkin reaction of pyridine and CH_3_I, we found that even in the polar acetonitrile solvent, the OEEF still lowers the energy barrier by ∼8 kcal/mol.^55^ Water or a mixture of acetonitrile-methanol as solvents may screen the OEEF more significantly and affect smaller barrier reduction.
f In using eq 1, the interaction energy will be stabilizing (negative) when the vectors F_Z_ and μ_Z_ are oppositely oriented (e.g., Figure 3). The directions of these vectors depend however on the conventions of the particular quantum chemistry (QC) package which one uses. We use the Gaussian convention, in which the head of the arrow of the OEEF is defined as the positive pole and the tail is negative. The same applies to the dipole moment (e.g., Figure 3).

I will now proceed to provide simple and useful rules for determining the reaction axis and will subsequently discuss my vision for 2050.

## 8 Applications of the Reaction Axis Rule

**Scheme 4:**
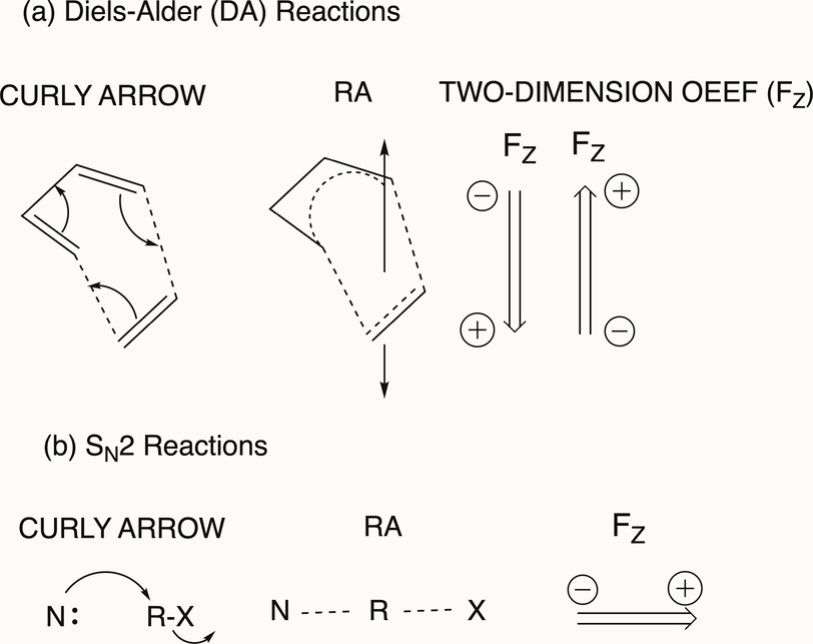
Curly Arrow Pushing, the Reaction Axis (RA), and the OEEF Direction (F_Z_) for: (a) a Diels-Alder (DA) Reaction and (b) an S_N_2 Reaction The doubly headed arrow in (a) indicates that the OEEF can in principle affect reactivity in both directions of the Z axis. The scheme was produced by the author of the present Perspective.

The “reaction axis” (RA) is *the direction along which the electron pairing of the reactants is converted to that of the products.* For a given reaction, this electron flow can be “traced” by use of the curly arrow pushing mnemonic as done in organic chemistry.^2−4^Scheme 4 shows two examples of the Diels–Alder (DA) and S_N_2 reactions.

**Figure 8:**
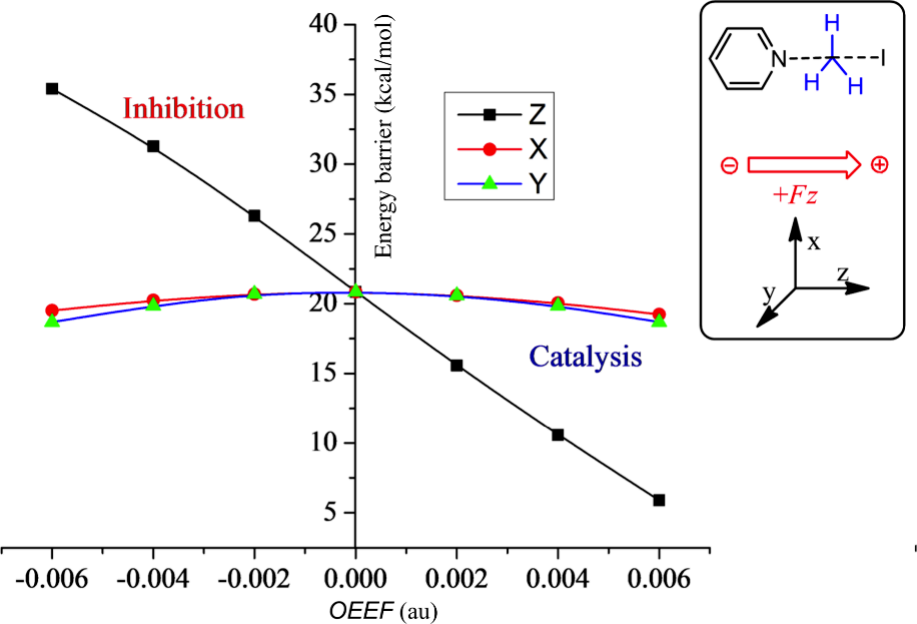
Impact of OEEFs on the Menshutkin S_N_2 reaction, along the X, Y, and Z directions. It is seen that F_Z_·> 0 leads to barrier lowering, while flipping the F_Z_ direction raises the barrier. OEEFs along the X and Y directions exhibit little or no effects. The Figure is adapted from ref (29). Copyright (2018) American Chemical Society.

The curly arrow pushing for the DA reaction in Scheme 4a shows that the RA is oriented in the direction which is perpendicular to the planes of the diene and dienophile. As such, the OEEF impact is localized along this axis, which indicates the flow of charge during the process.^3^ Indeed, as shown in Figure 3, the RA for cyclopentadiene reacting with maleic anhydride is along the Z axis. The other directions (X and Y) have little effects. The same applies to the S_N_2 reaction (see Figure 8 later).

Let me emphasize that in the DA reaction the electron flow is, in principle, two-directional along the Z axis. In one direction, the charge flows from the donor to the acceptor, i.e., from the HOMO of the diene to the LUMO of the dienophile.^28^ In the second, and usually less important direction, the flow is from acceptor to the donor, i.e., from the HOMO of the dienophile to the LUMO of the diene.^17,56^ As such, there can be in principle a two-way catalysis along the Z axis, as shown by Ramanan et al.^56^ This is likely to occur in nonpolar cycloaddition reactions. In DA reactions which involve good donor–acceptor pairs, reversing the OEEF encounters a significant energy rise to reach the conversion point.^56^ This is apparent from Figure 3a, which shows an energy rise of ∼8 kcal/mol in the region of F_Z_ > 0 (before reaching conversion).^28^ Otherwise, in a limited range of OEEFs (e.g., Figures 3 and 8), the plots of the energy barriers vs the OEEF visually appear as linear.

Scheme 4b shows the S_N_2 reaction, where the curly arrow promotes an electron flow from N to X through the R group. The F_Z_ that will enhance the rate of the S_N_2 reaction coincides with the direction of the corresponding electron flow in the RA. Figure 8 shows the computed OEEF impact on the Menshutkin reaction between pyridine and methyl iodide.^29^ It is apparent that F_Z_ affects the energy barrier dramatically, while F_X_ and F_Y_ have little if any impact on the reaction barrier.

This RA (for S_N_2) is representative of many atom/group transfer reactions, e.g., H atom abstraction by oxo-metal complexes (e.g., the active species of cytochrome P450 and nonheme enzymes and the corresponding synthetic models) from an alkane (e.g., Figure 2). Using curly arrow pushing will similarly generate the RAs for the elimination reaction in the E2 mechanism and for nucleophilic and electrophilic vinylic and aromatic substitutions. It is interesting to speculate about the site selectivity of nucleophilic reactions with substrates that possess two electrophilic sites. Will reactivity change as shown in Figure 2 for the reaction of compound I with propene and as reported in Figure 5 by Kanan et al.?^11^ What about the product selectivity reversal in the reaction reported by Matile et al?^7^

In summary, therefore, the application of OEEFs along the reaction axis will accelerate nonpolar reactions, control product selectivity by a flip of the field, control spin-state selectivity,^3,4^ mechanistic selection, and so on... Furthermore, the application of an OEEF serves as tweezers that orient molecules in space^52−54^ and make them react (along the reaction axis^53^). OEEF orientations “off the reaction axis” were found to control selectivity patterns and chiral discrimination in Diels–Alder reactions.^57^ The field’s direction affects similarly bonds, molecular structures,^1,3−5^ and solvent electro-freezing, e.g., of ammonia.^58^ Recently we showed that an oscillating OEEF (0.02 V/Å) can decompose irreversibly senile plaques more effectively than a static OEEF of the same intensity.^59^*Cis–trans* isomerization reactions of cumulenes were recently studied by Zang et al.^60^ Similarly, OEEF-stimulated isomerization of diazo-benzene derivatives was reviewed.^3^ As reflected by the currently available variety of OEEF techniques and topics,^1,61−64^ the area is expanding, and the possibilities may be innumerable.

## 9 A Vision of the Field in 2050: Scaling Up OEEF Reactions

Given the above potential, we envision that as techniques further mature, chemical syntheses and structural transformations may become exercises in applying OEEFs to oriented molecules and aggregates!

An essential future development of electric-field-dependent chemistry is the scaling up of experimental methods to molar quantities and the accommodation of the currently used OEEF equipment to the upscaling demands. In the absence of this capability, the area will remain an inspiring notion but one that is not necessarily practical. However, once experimental methods are scaled-up, this will bring the topic into main-stream chemistry, engage physical, organic, and inorganic chemists alike, and provide gentle means for chemical reactions (e.g., usage of lower temperatures, avoidance of undesired contaminant species due to usage of transition-metal catalysts^9^). Chemistry will change! This is an ideal vision for the field for 2050.

Let me elaborate on the scale-up issue for the various strategies of delivering electrostatic and electric fields to enhance reaction rates. Already in 1970, Pocker and Buchholz^18^ used huge concentrations of lithium perchlorate (∼6 M) in ether solutions and reported million-fold rate enhancements of classical physical organic chemical reactions. In this respect, the strategies of pH switching,^14^ or using, e.g., Li^+^, Cu^+^, or other ions to enhance reactivity^36,37^ can, in principle, be applied on quantitative molar scales. However, I say this with some reservation since no one has yet tried exploring/understanding **more thoroughly** the Pocker-Buchholz^18^ approach of using huge concentrations of the salt. Why it is so, still puzzles me!

Further development of this subfield of charging (either by means of adding salts^36,38^ or by pH-switching^14^) will require a thorough exploration of its practical potential and a derivation of the conceptual principles. Such developments will certainly cause this venue to flourish in the time left until 2050.

Let me try to focus the vision on reactions under OEEFs. The governing rules for applying OEEFs during chemical reactions were outlined above (e.g., in section 7). Furthermore, the rules were tested in some beautiful experiments, e.g., in Figures 4–6.^6,7,11^ Quantitative yields are a major issue with all these experiments which use OEEFs. Thus, the reactions are carried out either with single molecules (Figure 4) or with rather small concentrations of molecules as shown in Figures 5 and 6. As such, these experiments still constitute proofs of principle of the OEEF concept. An expansion to molar scales is required if one dreams of utilizing OEEFs for chemical syntheses. Since OEEFs lower the activation energy, most such chemical syntheses can be carried out at lower temperatures and avoid contaminants due, e.g., to usage of transition-metal catalysts.^9^ An effort to harness OEEFs for chemical synthesis is, therefore, highly attractive.

For example, improving the taste and fragrance of wine requires the addition of small amounts of esters. Esterification in the wine industry is catalyzed by various processes that leave behind undesired contaminants. To avoid these negative issues, Zhang et al.^9^ investigated the esterification of ethanol and acetic acid, using a moderate pulsed-electric-field (PEF), which lowered the energy barrier of the reaction by, e.g., 4.3 kcal/mol. This PEF-induced rate enhancement may either originate in an electric field effect or be due to a local thermal effect in the liquid or still to a combination of the two effects. Importantly, however, Zhang et al.^9^ used the PEF in **a continuous-flow system**. Such a system is, in principle, scalable to produce molar quantities. Will this become a standard method for reactions of liquids or in solutions in 2050?

The combination of a continuous-flow setup and employing molecular entities as the sources of OEEFs^8,42−44^ is another promising approach. Thus, for example, Zare et al.^42,43^ use water droplets, in which the O–H bonds on the surface of the droplet, as well as ions in the interface between droplets, deliver powerful OEEFs^10,65,66^ that catalyze unusual reactions, with efficiency that increases with decrease in the diameter of the droplets.^65^ As mentioned above, the droplets generate OEEFs, which catalyze nucleophilic cleavage reactions^10,48^ in accord with earlier calculations^29^ (see Figure 8). Currently, Zare’s method scales from micromoles to millimoles, but he hopes to scale it further up. Such upscaling of the water-droplet technique will bring it into main-stream chemistry and may change chemistry as we know it now.

**Figure 9:**
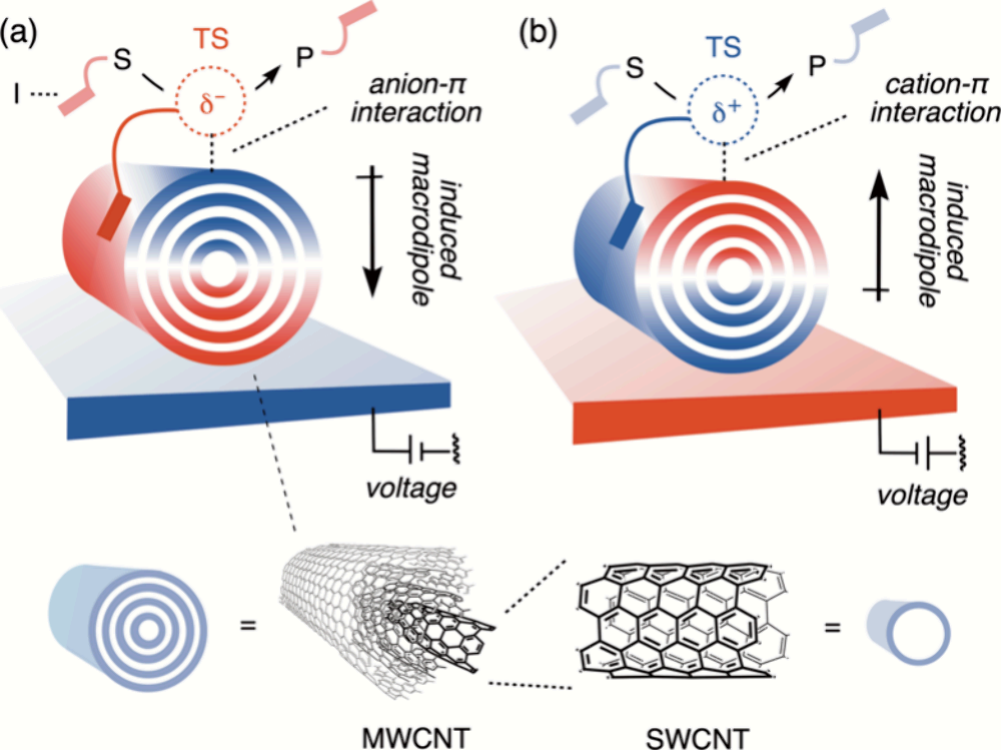
(a, b) Electric-field-induced catalysis in electromicrofluidic reactors, which involve MWCNTs. Applied voltages in opposite directions charge the surfaces that polarize the MWCNTs and induce oppositely oriented macrodipoles ((a) vs (b)) that generate corresponding OEEFs. The substrate (**S**) is deposited on the MWCNT surface using planar molecules like pyrene which is attached to substrate **S**. The OEEFs, due to the polarized MWCNT, enhance the rate of product (**P**) formation from **S**. Provided with courtesy of the lead author of ref (8), S. Matile. The Figure is reproduced with permission under a Creative Commons Attribution license from ref (8). Copyright (2023) American Association for the Advancement of Science.

**Figure 10:**
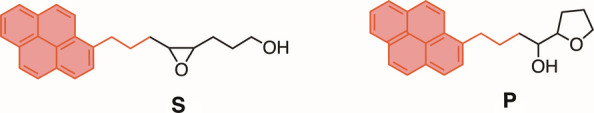
An example for **S** and **P** for the transformation of 3-hydroxo epoxide (**S**) to an ether molecule (**P**). The pyrene rings in **S** and **P** serve as “adhesives” which attach the reacting molecule (**S**) on the surface of the MWCNT (in Figure 9, the pyrenes are depicted as small rectangles). Provided with courtesy of S. Matile, the author of ref (8). The Figure is reproduced with permission under a Creative Commons Attribution license from ref (8). Copyright (2023) American Association for the Advancement of Science.

Most recently Matile et al.^8^ used reaction-cells with a continuous-flow and a built-in polarizable catalyst. The catalyst in Figure 9 is made of multiwalled carbon nanotubes (MWCNTs) that are highly polarizable, much more so than the single-walled unit (SWCNT). In turn, the highly polarizable MWCNTs generate the requisite OEEF that converts various substrates (**S**) to products (**P**). As shown in Figure 9a,b, the OEEF depends on the direction of the applied voltage that charges the surface underneath the MWNCTs. An example of a pair of **S** and **P** is shown in Figure 10.

Matile’s work was highlighted recently.^67^ In my discussion with him about his new approach, he was optimistic that this method^8^ will be up-scaled to serve as a practical synthetic method.

Other than the electric field intensity, my group showed recently^59^ that an oscillating OEEF (Os-OEEF) of 0.02 V/Å decomposes a senile plaque much more readily than does a static OEEF of the same intensity. Pulsed-Electric-Fields (PEFs)^9^ seem also to be very effective. We do not fully understand, at present, the origins for this superiority of the Os-OEEF and the effectiveness of PEFs. But as we are presently investigating these issues, an answer may emerge well before 2050.

Application of OEEF on multistep reactions will generally modulate the barriers for all of the steps. An interesting result for the enzyme tyrosine hydroxylase^68^ shows that the local electric field of the enzyme accelerates significantly the rate-determining step, the formation of the active species of the enzyme, while somewhat slowing down the product-forming step via aromatic hydroxylation. Generally, for catalytic cycles, one can conceptualize the OEEF effect on the energy span of the cycle,^69^ which is the rate-controlling barrier of the cycle.

Considering all the above developments and their prospects of up-scaling, it is apparent that the usage of OEEFs has the potential of shaping the future of chemical synthesis in organic, inorganic, and physical chemistry and, thereby, affecting the entire field of chemistry. At the very least, in 2050, OEEF usage will change chemical education, if not also the arts of chemical synthesis, structure control,^48,58,59^ and manufacturing of novel devices.^63^

### Avoiding Pitfalls

Before turning to summarize the Perspective, I wish to suggest some useful advice to readers who are interested in incorporating OEEF effects into their experiments but would like to avoid pitfalls. Occasionally, an experimental system may include acid/base catalysts or trace metal cations. In such a case, it is necessary to verify the contributions of OEEF effects vis-à-vis the effects by the above catalysts as done, e.g., in ref (37). The recommendation is to run quantum chemical (QC) calculations of the reaction system without any additives (as done in ref (37)) and compare the QC results to those obtained for the experimental system in the presence of the catalyst additives. Similarly, one may carry out OEEF calculations with and without the acid–base and metal ion catalysts. In general, prior QC tests are recommended as guides and as cautionary means in mechanistic interpretations.

## 10 Summary

I started this Perspective by revealing my obsession to understand the origins of the million-fold catalysis found by Pocker and Buchholz^18^ by use of highly concentrated LiClO_4_ (∼6 M) in ether. I still do not, but it is on my to do list! I then continued this style of story-telling for the rest of the Perspective including this summary.

My attempts to comprehend the impact of OEEFs on chemical reactivity and structure led me to formulate a set of guiding rules (like the reaction axis rule, the ability of OEEF to act as tweezers which juxtapose molecules for reactions, and the equation which provides the interaction of a field with a dipole moment, etc.). Quantum chemical computations, which were carried out in various groups,^3−5^ followed these guiding rules and demonstrated that OEEFs control reactivity and selectivity in a variety of reactions: H-abstraction, Diels–Alder reactions, S_N_2, and many more reactions, including the selection of the reactive spin states.^3,4^

I continued the story with verifications of specific predictions by experiments that generate in situ electric fields (e.g., STM single-molecular techniques, surface charging, usage of double layers, pH switching, usage of water droplets to deliver OEEFs, etc.). The turning point was the prediction of OEEF effects on the Diels–Alder reaction, which was verified by an elegant STM experiment,^6^ followed by other verifications.^12,13^ Since then, the approach of using electric fields to accelerate chemical reactions has been amply verified, thus, making the area attractive for experimental scrutiny and usage.

I ended the Perspective with methodologies that can be upscaled (e.g., using OEEFs and PEFs in continuous flow systems, water droplets, ionic additives, etc.). Once up-scaling is achieved, **this will change the nature of chemical synthesis in all areas of chemistry**, and may even play a role at some point in chemical industry. OEEF is a universal catalyst, and as such its adaptation looks promising.

Should upscaling attempts fail, the OEEF approach will nevertheless affect the conceptual fabric of chemical education. Be that as it may, the perusal of this technology will also bring about the developments of more elaborate and refined techniques to apply OEEF effects on structure and reactivity. This in a nutshell is my vision for electrified chemistry in 2050.

## Dedication

The paper is dedicated to Yitzhak Apeloig on the occasion of his forthcoming 80th birthday.
